# Machine Learning Establishes Single-Cell Calcium Dynamics as an Early Indicator of Antibiotic Response

**DOI:** 10.3390/microorganisms9051000

**Published:** 2021-05-05

**Authors:** Christian T. Meyer, Megan P. Jewell, Eugene J. Miller, Joel M. Kralj

**Affiliations:** BioFrontiers and MCDB Department, University of Colorado Boulder, Boulder, CO 80303, USA; christian.meyer-1@colorado.edu (C.T.M.); megan.jewell@colorado.edu (M.P.J.); eugene.miller@colorado.edu (E.J.M.)

**Keywords:** calcium, *E. coli*, aminoglycosides, polymyxin B, antibiotic resistance, machine learning

## Abstract

Changes in bacterial physiology necessarily precede cell death in response to antibiotics. Herein we investigate the early disruption of Ca^2+^ homeostasis as a marker for antibiotic response. Using a machine learning framework, we quantify the temporal information encoded in single-cell Ca^2+^ dynamics. We find Ca^2+^ dynamics distinguish kanamycin sensitive and resistant cells before changes in gross cell phenotypes such as cell growth or protein stability. The onset time (pharmacokinetics) and probability (pharmacodynamics) of these aberrant Ca^2+^ dynamics are dose and time-dependent, even at the resolution of single-cells. Of the compounds profiled, we find Ca^2+^ dynamics are also an indicator of Polymyxin B activity. In Polymyxin B treated cells, we find aberrant Ca^2+^ dynamics precedes the entry of propidium iodide marking membrane permeabilization. Additionally, we find modifying membrane voltage and external Ca^2+^ concentration alters the time between these aberrant dynamics and membrane breakdown suggesting a previously unappreciated role of Ca^2+^ in the membrane destabilization during Polymyxin B treatment. In conclusion, leveraging live, single-cell, Ca^2+^ imaging coupled with machine learning, we have demonstrated the discriminative capacity of Ca^2+^ dynamics in identifying antibiotic-resistant bacteria.

## 1. Introduction

Calcium (Ca${}^{2+}$) is a critical ion for eukaryotic cells connecting membrane voltage to cellular signaling [1]. From classic experiments in the squid giant axon [2] to plant immune signaling [3], the role of Ca${}^{2+}$ in voltage-mediated cell signaling is universal to life. However, compared to eukaryotic systems, the extent to which Ca${}^{2+}$ dynamics modify cell behavior in prokaryotes is only just being discovered [4,5,6,7]. Recent evidence has connected Ca${}^{2+}$ in prokaryotes to diverse processes including oxidative stress [8], motility [9], virulence [10], and chemotaxis [11]. Additionally, Ca${}^{2+}$ is used for mechanosensation in *Escherichia coli* [12], suggesting that the role of Ca${}^{2+}$, as well as sodium and potassium, in cell–cell communication is not limited to eukaryotic organisms [13,14,15,16,17]. However, while Ca${}^{2+}$ binding domains have been identified in prokaryotic genomes ([18,19,20,21,22,23,24] and reviewed in [4,5]), it remains challenging to prove the functionality of these domains.

Nevertheless, despite its ubiquity, Ca${}^{2+}$ represents a Promethean paradox for cells, being both necessary for life, but also cytotoxic, a dynamic signaling molecule necessarily kept within a narrow concentration range [25,26]. Ca${}^{2+}$-dependent cytotoxicity occurs both from the complexation of calcium phosphate [27] as well as signaling dependent mechanisms. In eukaryotes, these signaling mechanisms include Ca${}^{2+}$-dependent proteases [28], phospholipases [29], endonucleases [30], and activation of apoptotic proteins [30]. To precisely regulate intracellular Ca${}^{2+}$ levels, eukaryotes utilize a complement of Ca${}^{2+}$ transport proteins including ATP-dependent pumps, voltage-gated membrane channels [31], ion porters, and sequestration into organelles such as the endoplasmic reticulum, mitochondria, and lysosomes [32,33]. That such a wide array of regulatory mechanisms have evolved to maintain and modulate intracellular Ca${}^{2+}$ levels, and initiate programmed cell death in the event of failure, suggests that Ca${}^{2+}$ toxicity is a persistent danger in the life of a cell. In prokaryotes, conversely, only recently has a native voltage-dependent Ca${}^{2+}$ channel been discovered [34]. The extent to which similar mechanisms exist in prokaryotes for modifying Ca${}^{2+}$ pools and initiating cell death is yet undetermined. Regardless, the double-edged sword of Ca${}^{2+}$ is an unavoidable evolutionary constraint for single-cell organisms.

Recent evidence demonstrated that Ca${}^{2+}$ transients (characterized by hyper-oscillations in intracellular free-Ca${}^{2+}$) were sufficient and necessary for the bactericidal activity of aminoglycosides in *E. coli* [35]. This is in contrast to the previous claims that voltage-dependent drug uptake [36] or reactive oxygen species (ROS) [37,38] are central to cell death initiated by aminoglycosides. Other ions, in particular Mg${}^{2+}$, have been shown to mediate the response of *Bacillus subtilis* to other translation inhibitors [39]. These studies raise the possibility that ionic imbalance is a common mechanism of cellular death induced by bactericidal antibiotics [40].

Because the maintenance of Ca${}^{2+}$ is integral to cellular health, we hypothesized that Ca${}^{2+}$ disequilibrium is an early marker of antibiotic activity. In this work, we test this hypothesis by leveraging a genetically encoded fluorescent biosensor of free Ca${}^{2+}$ [41,42] to record Ca${}^{2+}$ dynamics at single-cell resolution. We find Ca${}^{2+}$ transients are a dose-dependent and time-sensitive marker of aminoglycoside activity which mirror the number of viable cells. Using a machine learning framework, we find the antibiotic-induced Ca${}^{2+}$ signature precedes aberrant cell growth as well as reliably distinguishes kanamycin sensitive and resistant cells. Applying our framework to a panel of antibiotic compounds, we find Ca${}^{2+}$ dynamics encode drug action in more drug classes than just aminoglycosides. In particular, we find a strong temporal Ca${}^{2+}$ signature in response to Polymyxin B, a broad-spectrum antibiotic for Gram negative bacteria. In summary, by combining machine learning with direct measurements of Ca${}^{2+}$ activity in single cells, we present evidence of Ca${}^{2+}$ as an early signal of antibiotic response.

## 2. Materials and Methods

### 2.1. Experimental Set Up

**Figure 1:**
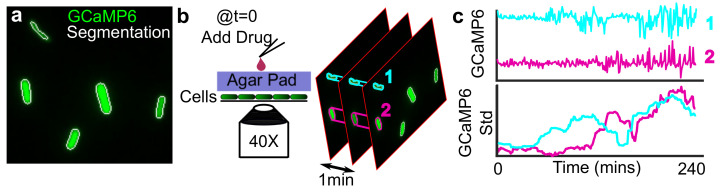
*Measuring Ca*${}^{2+}$*dynamics in single E. coli.* (**a**) BW25113 cells are labeled with a GCaMP6f-mScarlet dual fusion plasmid (mScarlet channel not shown). Cells are segmented (white outline) using both channels (see Methods *Image Processing*). (**b**) Dilute cells in log-phase growth are affixed to a 2% agarose pad and inverted into a 96-well plate for imaging. Drug addition is done at t = 0 by pipetting on top of the agarose. Cells are imaged at 1-minute intervals. (**c**) The Ca${}^{2+}$ dynamics are extracted from the single-cell segmentation (top panel). Rapid oscillations in the cellular Ca${}^{2+}$ content, so-termed Ca${}^{2+}$ transients, have previously been observed in response to aminoglycosides [35]. To quantify these oscillations, a moving window standard deviation (std, bottom panel) is calculated. As the magnitude of the Ca${}^{2+}$ transients increases in a given time-window, the std increases. The sliding window size is 30 min.

*E. coli* strains BW25113 and mntH (genomically-integrated kanamycin-resistant) were acquired from the Yale Coli Genetic Stock Center. Cells were transformed, as previously described [35], with a GCaMP6-mScarlet containing plasmid (Addgene #158979) (Figure 1a). *B. subtilis* strain with genomically-incorporated, isopropyl-β-d-thiogalactopyranoside (IPTG)-inducible expression of GCaMP6f (hyperspank promoter) was obtained from Dr. Ethan Garner (Harvard University). The genomically incorporated cassette in *B. subtilis* also included spectinomycin resistance. For each experimental run, three GCaMP6 expressing colonies were picked from an agar plate and grown overnight in LB 37 C with shaking between 150 and 200 rpm and with carbenicillin (100 μg/mL) for *E. coli* and spectinomycin (100 μg/mL) for *B. subtilis* to maintain GCaMP6 expression. From overnight cultures, cells were diluted 1:100 in PMM minimal media (pH 7.5) and grown at 30 C for 2 h shaking (150–200 rpm). The PMM recipe used is: 1x M9 salts (Sigma), 0.2% glucose (Sigma), 0.2 mM MgSO4, 100 M CaCl2, 1x MEM amino acids (Gibco). For inducing the GCaMP6 expression in the *B. subtilis* cells, IPTG was diluted to 1 mM concentration in both the LB and PMM media.

After 2 h of outgrowth in PMM, 2 L of dilute cell culture was added to the top of 200 L 2% low melt agarose pads, molded to fit in 96-well square glass-bottom plates (Brooks Automation, MGB096-1-2-LG-L) (Figure 1b). *B. subtilis* required concentrating 10X by centrifugation before plating. See [35] for details regarding well molds and pad preparation. For experiments involving PI (3 μg/mL), CCCP (50 M), EGTA (5 mM), and excess Ca${}^{2+}$ (1mM), reagents were diluted directly in liquid agarose before pouring the pad. Concentrations of free Ca${}^{2+}$ in the presence of EGTA were calculated according to [43]. For *B. subtilis* experiments, IPTG was also included in the pad at 1 mM concentration. After 15 min, the pad with affixed cells was inverted and pressed into the bottom of a pre-warmed imaging plate (30 C). Imaging positions were selected on the microscope and then, after the cells had been on the pad for exactly 1 h at 30 C, 5 L of 40X drug was added to the top of the pad, and the imaging was initiated immediately. Measurements with a fluorescent dye show compounds diffuse through the agarose with pharmacokinetics which better mimic drug uptake *in vivo* (Figure S1). Imaging experiments were conducted at pH 7.5 and without carbenicillin or spectinomycin. Each experiment consisted of selecting 3 colonies (biological replicates) that were imaged in separate wells.

Imaging took place using a Nikon Ti inverted microscope running the Slidebook software package (3i Inc). Fluorescent excitation was achieved with a laser source (488 nm and 561 nm) using a high-angle illumination to minimize the out-of-focus background. The excitation laser was brought to a focus at the back aperture and then a pair of galvo mirrors (3i Inc) was used to rotate the image around the back focal plane of the objective, minimizing sample-induced striping. All images were acquired with either a 40x, NA 0.95 or 60x, NA 0.95 air objective. Images were acquired on two sCMOS cameras (Photometrics, Prime 95B) using a 570 nm beam splitter (Cairn, TwinCam) to image mScarlet and GCaMP6 channels separately. Images were acquired at 1-minute intervals for 240 time-points. Each experiment contained biological triplicate of the tested conditions distributed between 50 and 70 fields of view.

### 2.2. Image Processing

The Ca${}^{2+}$ dynamics were extracted by segmenting single cells (white outline, Figure 1a) using a Hessian-based routine specific for identifying tubular structures. Image processing was done in Matlab (Mathworks, R2020a) and followed the general scheme described in [35]. Briefly, the illumination profile for all wells in an experimental run was estimated from the average of 50 images per well. Morphological opening and blurring were used to broaden the illumination pattern before correcting the images. After illumination correction, the jitter in the movie was removed by aligning each sequential frame using a fast 2D Fourier transform implemented in Matlab. Once images were illumination-corrected and aligned, the background was estimated for each frame using morphological opening and subtracted from the original image.

**Figure 2:**
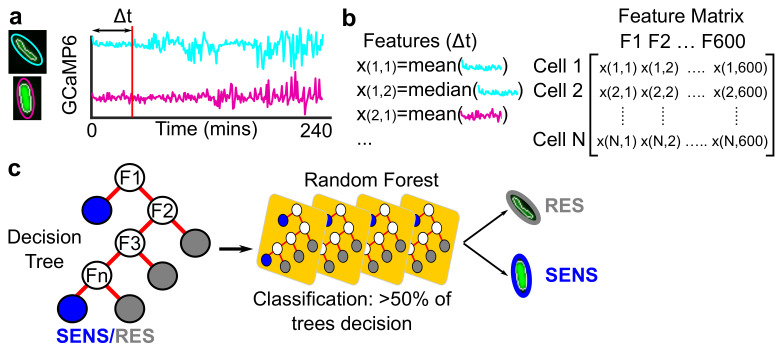
*Machine learning schematic to measure how Ca*${}^{2+}$*dynamics distinguish sensitive and resistant cells at single*-*cell resolution.* (**a**) For each cell’s Ca${}^{2+}$ trace, summary statistics are calculated. (**b**) Features are different summary statistics (i.e., moments) of the Ca${}^{2+}$ trace with different smoothing functions and normalization protocols resulting in 600 total features, (see Supplementary Materials section *Feature calculation from fluorescent traces*). We define a value Δt corresponding to the length of the trace to subset before calculating the features. Calculating these features for a particular Δt for all cells results in a feature matrix. The feature matrix is used to train a random forest classifier to distinguish sensitive and resistant cells. (**c**) The random forest is comprised of decision trees (**left**), which make a binary classification for each cell based on the calculated trace features (Fn). Many decision trees are combined to create a random forest, which makes a final classification by weighting the decision from all the trees. As Δt increases, more of the Ca${}^{2+}$ trace is included, improving the classifier’s discriminative capacity. Therefore, the random forest acts as a stopwatch for how early the sensitive and resistant cells can be reliably distinguished.

Segmenting cells was done using the Hessian-based *fibermetric* segmentation routine implemented in Matlab. Segmented regions were included only if they met a minimum area and intensity threshold. This segmentation was done for both the mScarlet and the GCaMP6 channels before adding the segmentation maps together to get the final binary mask. To simultaneously measure cell growth and Ca${}^{2+}$ dynamics, the segmentation map was redrawn such that each segmented object was enclosed by an ellipse with a major and minor axis equal to 1.0 and 1.5X the major and minor diameter of the segmented cells, respectively, (Figure S2). For segmenting *B. subtilis*, the ellipse axes were equal to 0.6 and 0.8X the major and minor diameter of the segmented cells, respectively. We found these values to faithfully capture cells growing and drifting in the wide array of conditions we tested (Videos S1 and S2). The mean, standard deviation (std), minimum, and maximum GCaMP6 and mScarlet signals within the enclosed ellipse for each time-point were extracted from the pixels enclosed within each ellipse (Figure 2a). Within each ellipse, only pixels with a signal greater than background were included in the summary statistics. We refer to these summary statistics collectively as the Ca${}^{2+}$ “trace”. The change in cell area over time was also computed by counting the number of non-background pixels inside the ellipse for each frame.

### 2.3. Machine Learning Classifier

To investigate the temporal information in the Ca${}^{2+}$ dynamics, we leveraged a random forest classifier trained to distinguish sensitive and resistant cells at different time intervals (Δt) (Figure 2). Specifically, we trained the random forest using features calculated from the Ca${}^{2+}$ traces (Figure 2a,b). Data cleaning steps were as follows. Mean GCaMP6 intensity was cleaned by filling any missing values with a moving mean (window 15 frames) or with the nearest non-missing value. Additionally, any cells where the cell drifted out of the ellipse were identified and removed by identifying frames where the cell area dropped to zero. Finally, outliers were removed from the data using multi-dimensional gating based on the segmentation area, major/minor axis lengths, circularity, and length of the perimeter. The mean Euclidean distance in multi-dimensional space for each cell to all other cells was computed, and cells in the 98th percentile or above in average distance from all other cells were excluded. To compensate for the less accurate segmentation in the *B. subtilis*, we removed cells in the 85th percentile or above in the multi-dimensional gating.

Once the data was cleaned, the feature matrices were computed (Figure 2b). A total of 600 features were calculated for each trace regardless of trace length. Features included convolutions of different sizes to increase the long-range information embedded in the trace. Feature descriptions are included in the Supplementary Materials *Feature calculation from fluorescent traces*. The feature matrix was normalized and any features or cells which were undefined were removed. Features were further reduced using Principal Component Analysis (PCA) to the minimal set of eigenvectors comprising >95% of the variance in the dataset. For the GCaMP6 signal in the kanamycin treated *E. coli* dataset, this reduced the dimensionality to 58 features.

To identify optimal hyper-parameters for each treatment condition, the full time-course (Δt = 240 min) was split 80/20 into training and testing data sets. The training data was fit to a random forest classifier using Matlab’s *fitcensemble* method (Figure 2c) in 2 stages. During the first stage, the hyper-parameter sweep optimized the boosting function, while in the second stage, the learning rate, maximum number of splits, minimum leaf size, and number of learning cycles for a particular boosting function were optimized. Both boosting function and learner parameters optimization used 200 learning iterations. We consistently found that the AdaBoostM1 method [44] to be the optimal boosting approach. Boosting is a technique that continuously updates the weights in the dataset as decision trees are being added to the random forest to “boost” the classifier performance on samples that are poorly performing in early trees. The AdaBoostM1 method was used for all drugs; however, the optimal hyper-parameters for each drug were determined independently. See Table S1 for the hyper-parameters for each drug.

To classify sensitivity and resistance based on cell area, we use Support Vector Machines (SVM), which are appropriate for distinguishing classes in univariate datasets. The same approach was used for selecting hyperparameters based on first maximizing the full-time-course classifier performance. Hyperparameters optimized include the kernel density and box constraints. These hyperparameters were then used for training classifiers with different Δt. An SVM was also used to measure the performance of only Ca${}^{2+}$ transients (Figure 1c) in predicting sensitivity and resistance.

Based on the optimal hyper-parameters for the full-time-course dataset, the classifier performance for varying Δt was computed. Importantly, the PCA dimensionality reduction was performed on each Δt classifier separately. An equal number of sensitive and resistant traces were used to train each Δt classifier to appropriately weigh the algorithm performance. Untreated sensitive cells were labeled as resistant to prevent the classifier from identifying the subtle cell-line specific differences in GCaMP6 expression. Classifier performance was measured using the area under the receiver operator curves (ROC AUC), as well as the error rate. Ten-fold cross-validation was performed to ensure classifiers were not being over-trained.

All code and processed data required to create and train the classifiers are available on BitBucket at https://meyerct1@bitbucket.org/meyerct1/gcamp_paper.git accessible as of 1 May 2021.

### 2.4. CFU Assays

CFUs were measured by plating treated cells onto LB-agarose without antibiotic and counting growing colonies. CFU measurements were conducted to mimic the experiments performed via microscopy. Briefly, three biological replicates were grown overnight in LB and diluted 1:100 in 5 mL PMM followed by growth at 30 C for 2 h. Antibiotic was added directly to liquid cultures after the outgrowth phase. The liquid cultures were repeatedly sampled after each predetermined treatment interval and plated. Plating the cells was done by first washing in PBS and diluting in a 10X series. From each of the 10X dilutions, 5 L was plated onto an LB agar pad and left to dry. Colonies were counted manually following incubation overnight at 37 C. The smallest number of CFUs observable in our assay is 200 CFUs/mL. To increase our limit of detection at 100 μg/mL kanaymcin after 2 h, 100 L treated culture was plated onto a 10 cm dish and counted to measure CFUs per milliliter. This experiment concluded that there are an estimated 30 CFUs/mL after 2 h of treatment compared with 3 ×$10^{7}$ CFUs/mL before treatment. Therefore, we expect approximately one cell in every million cells for this condition is viable.

## 3. Results

### 3.1. Ca${}^{2+}$ Transients Are a Dose- and Time-Dependent Indication of Sensitivity to Aminoglycosides, Preceding Changes in Cell Growth, in a Bacterial Population

**Figure 3:**
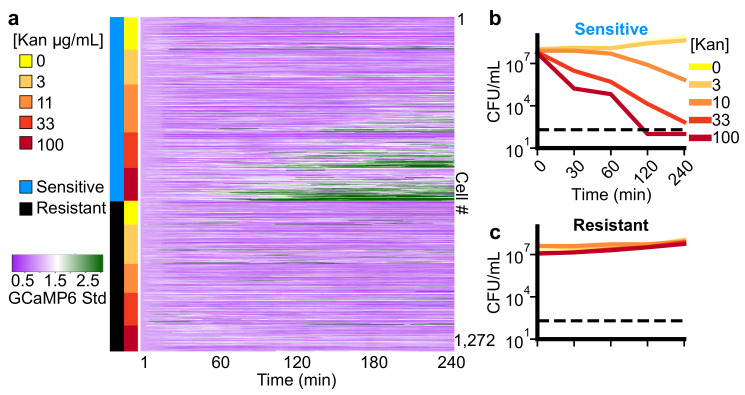
*The onset of Ca*${}^{2+}$*transients distinguishes sensitive and resistant cells and is dose and time dependent.* (**a**) Heatmap of 1272, single-cell, GCaMP6 std traces (see Figure 1) for sensitive (naive BW25112 cells, blue rows) and Kan resistant (mntH cells, black rows) *E. coli* treated with different concentrations of kanamycin (second color column; yellow->red). Green shading corresponds to high GCaMP6 std (colorbar) and therefore marks the onset of Ca${}^{2+}$ transients. (**b**,**c**) The colony forming units (CFUs) per mL for different exposure periods and concentrations of kanamycin for sensitive (**a**) and resistant (**b**) cells. Colors match the concentration colors in panel a. The limit of detection for our assay is 200 CFUs/mL (dotted black line, see Methods). Values for each concentration and exposure time are the average of 3 biological replicates.

A previous study demonstrated that Ca${}^{2+}$ transients, as measured by an increase in the moving standard deviation of GCaMP6 intensity over time (Figure 1c), distinguished *E. coli* cells, which permanently arrest from those that regrew in response to aminoglycosides [35]. Building on this observation, we first investigated how the time to onset of these Ca${}^{2+}$ transients related to the dose of kanamycin (Figure 3a). Titrating the concentration from 0 to 100 μg/mL (yellow to red, Figure 3a), we observed the fraction of cells experiencing Ca${}^{2+}$ transients (green in heat map) is dose-dependent for aminoglycoside-sensitive cells (blue colorbar). In the 100 μg/mL condition, most cells displayed sustained Ca${}^{2+}$ transients by 120 min from drug addition. Each successively lower dose saw Ca${}^{2+}$ transients with a temporal delay compared to the 100 μg/mL condition in a decreasing portion of cells. There was a notable absence of transients in the 3 μg/mL condition, indicating this concentration was well tolerated by the cells. These observations suggested that the time-to-onset and probability of Ca${}^{2+}$ transients were dose-dependent. Cells with a genomically encoded kanamycin resistance (resistant cells, Figure 3a, black colorbar) did not show evidence of GCaMP6 transients as expected. To investigate how the duration and concentration of kanamycin treatment altered the number of viable cells, we measured the colony-forming units (CFUs) across the same concentration range for exposures ranging from 30 to 240 min for both sensitive (Figure 3b) and resistant (Figure 3c) cells. We found a $10^{6}$ fold reduction in viability after 120 minutes exposure to 100 μg/mL kanamycin, which matches the concentration and time-point at which persistent Ca${}^{2+}$ transients are observed in the single-cell traces (Figure 3a). We also observed no significant change in the CFUs between the 3 μg/mL and untreated conditions at 2 h, corroborating our observation that the absence of Ca${}^{2+}$ transients is a proxy for viable cells [35]. Therefore, Ca${}^{2+}$ transients distinguished sensitive and resistant populations, and they mirrored the dose and time-dependence on viability during kanamycin treatment.

**Figure 4:**
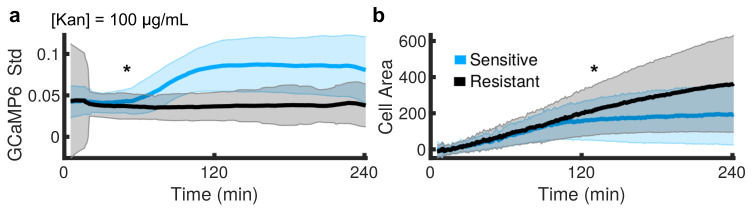
*The onset of Ca*${}^{2+}$*transients precedes measurable growth defects at the population level in response to kanamycin.* (**a**,**b**) Average (dark line) of single cell measurements for GCaMP6 std (**a**) and cell area (**b**) over time in 100 μg/mL of kanamycin. The squared variance between single-cells for each time-point shown in shaded error bar. The sensitive (blue, 5011 total cells) and resistant (black, 3005 total cells) populations diverge in 50 min by Ca${}^{2+}$ transients (**a**) while the divergence in cell growth (**b**) occurs at 131 min (asterisk, KS-statistic > 0.25).

We next compared the onset of Ca${}^{2+}$ transients to changes in cell growth between sensitive and resistant cells (Figure 4). At the population level, divergence in GCaMP6 std was observed in less than 50 min (asterisk, Figure 4a, KS-statistic > 0.25), whereas the area did not diverge until 131 min (asterisk, Figure 4b, KS-statistic > 0.25). This demonstrates that drug-induced alterations in Ca${}^{2+}$ homeostasis preceded changes in growth rate at the population level during kanamycin treatment.

### 3.2. Machine Learning Reveals Single-Cell Ca${}^{2+}$ Dynamics Are an Early and Reliable Marker of Kanamycin Activity

To quantify the minimum time required for Ca${}^{2+}$ dynamics to discriminate between sensitive and resistant cells, we constructed a machine learning framework to function as an in silico stopwatch. This framework took as input GCaMP6 traces from single cells that were labeled as either sensitive or resistant and trained a random forest classifier (Figure 2). The quality of the classifier was measured by predicting the identity of a new cell based on its GCaMP6 trace. The confidence in classifying the two populations depended on the length of the GCaMP6 trace used to train the classifier. This characteristic length we term Δt. For each cell, we calculated a feature matrix (Figure 2b) which depended on Δt. As Δt increased, more of the cell’s Ca${}^{2+}$ dynamics are included in the classifier reducing the classification error. We defined sensitive cells to be the BW25113 cells at 100 μg/mL. We defined resistant cells as either mntH cells (genomically-integrated KanR) treated at 100 μg/mL or untreated BW25113 cells. Labeling both the untreated BW25113 and treated mntH cells in our training data as resistant was critical to preventing the classifier from identifying subtle cell line differences in GCaMP6 expression rather than differences in Ca${}^{2+}$ dynamics. To benchmark the Ca${}^{2+}$ signal, we compared it to classifiers based on cell area and the mScarlet signal (see Methods). As the mScarlet signal integrates changes in protein translation, degradation, and cell growth, we consider it a proxy for estimating global phenotypic changes in the cell.

**Figure 5:**
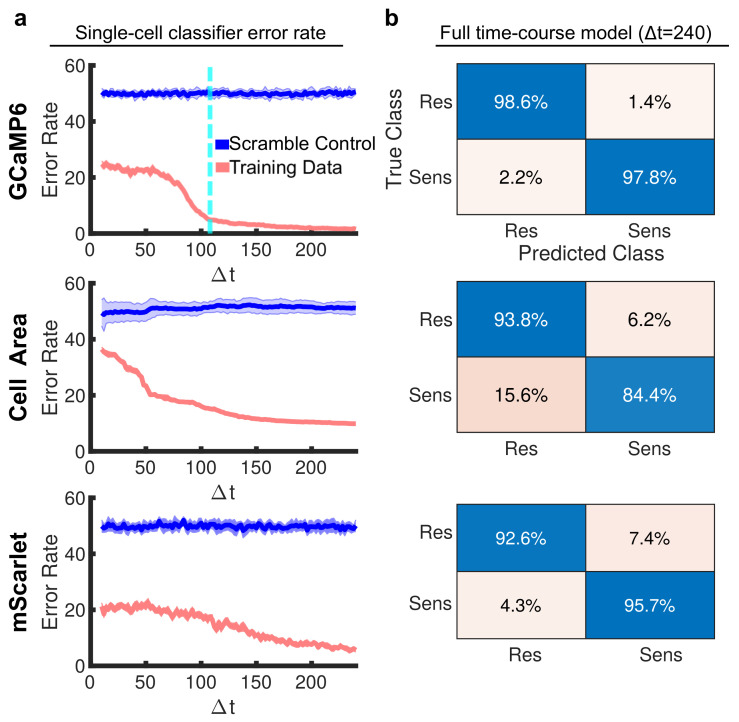
*Using machine learning to discriminate kanamycin sensitive from resistant cells at single*-*cell resolution.* (**a**) Comparing the error rate of machine learning classifiers as a function of Δt for GCaMP6, cell area, and mScarlet traces. A random forest classifier with AdaBoostM1 boosting (Figure 2) was used for the GCaMP6 and mScarlet traces while an SVM was used for cell area (see Methods). As the amount of the trace included in training the classifier increases (i.e., Δt increases), the total error rate of the classifier decreases (red curve). The hyper-parameters are the same for all Δt classifiers. The blue curve is the error rate of the same random forest architecture trained on scrambled cell labels. As expected, the classifier cannot improve upon a 50/50 guess when the traces are scrambled. This demonstrates the information content is unique to the Ca${}^{2+}$ dynamics. Shaded error bars are the standard deviation in the error rate calculated between 10-folds (80/20 split). Dashed, cyan line marks when the error rate for GCaMP6-based classifier is first below 5% (108 min). Neither cell area nor mScarlet signals achieve a <5% error rate in single-cell classification. (**b**) Confusion matrices for the full time-course classifiers based on the GCaMP6 trace (**top**), cell area (**middle**), and mScarlet trace (**bottom**). The confusion matrix was calculated based on a 20% subset of all cell traces withheld from the training dataset. Training used an 80/20, 10-fold cross-validation scheme. A total of 7557 and 4969 resistant (Res) and sensitive (Sens) single-cell traces were used for testing and training.

As expected, we found a strong temporal signature in the error rate (Figure 5a, top panel, red curve) for classifiers trained on GCaMP6 traces while cell area and mScarlet had less pronounced temporal dependence (Figure 5a, middle and bottom panel). Before the sustained onset of the Ca${}^{2+}$ transients (~120 min, Figure 3a), the error in the GCaMP6-based classifier began to decrease as the classifier begins to correctly discriminate sensitive and resistant cells achieving <5% error rate by 108 min (Figure 5a, top panel, dashed cyan line). When the labels were scrambled, the classifier cannot improve from a 50% error rate or equal to a random guess (Figure 5a, blue curve). The ~25% error rate of the GCaMP6-based classifier even at small Δt was a result of the classifier correctly distinguishing resistant cells at early time points, but unable to correctly classify sensitive cells any better than a random guess. Because the number of sensitive and resistant cells that feed into the classifier is equal, this leads to the ~25% error rate. Cell area and mScarlet based classifiers did not reach <5% error (Figure 5a, middle and bottom panels) within the 4-h measurement window. Finally, we compared the performance of a SVM classifier trained only on Ca${}^{2+}$ transients (std GCaMP6, Figure 1c) to our more complex random forest model and found that the inclusion of additional features substantially reduced the time to correctly distinguish sensitive and resistant cells (Figure S3). Therefore, Ca${}^{2+}$ dynamics are an early marker of kanamycin activity which preceded changes of several gross phenotypic modifications.

The overall discriminative capacity of Ca${}^{2+}$ dynamics for classifying single cells was compared to mScarlet and cell area using the full 240 min time-course. Classifying based on GCaMP6 results in an extremely accurate classifier (Figure 5b, top panel). Overall, the classifier had <3% error in classifying both sensitive and resistant cells, while mScarlet and cell area classifiers had greater error rates (Figure 5b, middle and bottom panel). In the case of cell area, even with the full time-course, up to 16% of the sensitive cells were misclassified as resistant (Figure 5b, middle panel). Ca${}^{2+}$ dynamics also outperformed mScarlet and cell area-based classifiers in other metrics of machine learning classifier performance (Figure S4). Interestingly, our original definition of Ca${}^{2+}$ transients (moving window std of GCaMP6, Figure 1b) was one of the most predictive features learned by the GCaMP6-based classifier (Figure S5) emphasizing the random forest was correctly learning to distinguish the sensitive and resistant cells based on observable changes in the Ca${}^{2+}$ dynamics.

We also tested our random forest framework at detecting the response of a Gram-positive bacteria (*B. subtilis*) to kanamycin. While our segmentation algorithm was not as efficient in detecting single *B. subtilis* cells (Figure S6a), the classifier was able to distinguish treated and untreated cells in as little as 52 min with <5% error (Figure S6b). Overall discriminative capacity of Ca${}^{2+}$ dynamics in this cell line was high (Figure S6c), but the most important feature was different than for the *E. coli* classifier (compare Figure S5a,b and Figure S6d,e). Specifically, the minimum GCaMP6 signal over time was the most predictive feature which we found significantly distinguished treated and untreated cells (Figure S6f, Video S2). Therefore, our approach is flexible enough to account for the varying reactions of different cell types to the same antibiotic; however, these results emphasize the need to train cell type-specific classifiers.

**Figure 6:**
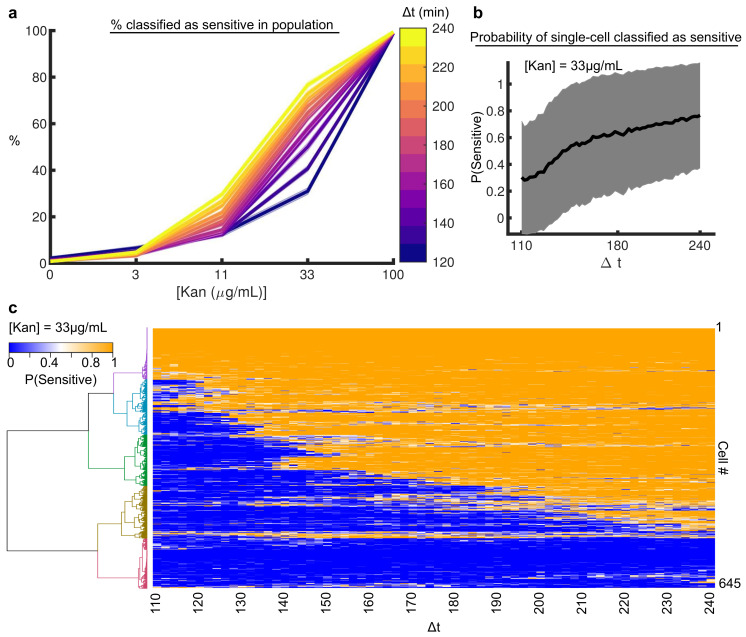
*Machine learning*-*based single*-*cell pharmacokinetics and pharmacodynamics.* (**a**) Percent of sensitive cells (*y*-axis) classified by the random forest for each concentration of kanamycin (*x*-axis) as a function of Δt (colorbar). Minimum Δt was 110 min corresponding to when the random-forest classifier’s error in classifying sensitive and resistant in the 100 μg/mL kanamycin condition was <5% (Figure 5b). As expected, the classifier correctly predicts ~0% of cells are sensitive at 0 μg/mL kanamycin as all untreated cells look resistant. At the 100 μg/mL kanamycin condition, ~100% are classified as sensitive. For the intermediate doses, as Δt increases (purple to yellow), the percent of cells classified as sensitive increases (*y*-axis). Shaded error bar is the standard deviation in percentage between 10 classifiers. (**b**) The average probability of a single cell at the 33 μg/mL concentration being classified as sensitive as a function of Δt. Shaded error bars denote the standard deviation in single-cell probability at every Δt. The large error bars are indicative of heterogeneity in single-cell predictions. (**c**) Heat map of a single-cells’ probability of being classified as sensitive (blue 0%, orange 100%) as a function of time (Δt, *x*-axis) for the 33 μg/mL kanamycin condition. The 645 cell probability traces are clustered (ward linkage) into 5 groups. Probability is calculated as the mean of 10 classifiers each built with randomly sampled 80% of the total data.

We further explored the observation that cell viability is dose- and time-dependent (Figure 3b) at the single-cell resolution using our random forest classifier. Specifically, we examined the pharmacodynamics (i.e., percent of cells affected) and pharmacokinetics (i.e., time to effect onset) as a function of time and dose. Beginning with GCaMP6-based random forest classifiers Δt>110 min (where the classifier error on the 100 μg/mL condition is <5%), we calculated the predicted fraction of sensitive cells at different doses (Figure 6a). As expected, we observed ~0% of the cells are classified as sensitive for all Δt values at 0g/mL condition and ~100% in the 100 μg/mL condition. The classification of resistance for the 0 μg/mL condition is because the untreated cells’ Ca${}^{2+}$ dynamics are indistinguishable from resistant cells. At intermediate doses, there is a marked temporal dependence on the fraction of sensitive cells. In particular, only ~30% of cells were classified as sensitive for the 33 μg/mL condition after 110 min, but by 240 min, over 70% were sensitive (Figure 6b).

However, all cells did not follow this average trend. Clustering single-cell probabilities over time, we found 5 classes emerged (Figure 6c). The classes corresponded to different onset times (gold, green, and blue clusters), already classified as sensitive at t = 110 min (magenta cluster), and never sensitive by 240 min (red cluster). The heterogeneity in the single-cell onset time suggested the pharmacokinetics of drug delivery at this dose is critical for determining cell killing, as was observed in the CFU assay (Figure 3b). At this dose, both variability in the time to drug action and the probability of drug activity played a role in determining the sensitivity profile of these cells. Remarkably, once a cell was predicted to be sensitive, the subsequent classifiers were consistent as Δt increased (i.e., more information was included). This provided a novel view of how the single cell activity of aminoglycosides depends on time and dose.

### 3.3. Ca${}^{2+}$ Dynamics Resolves Multiple Phases of Polymyxin B Activity

**Figure 7:**
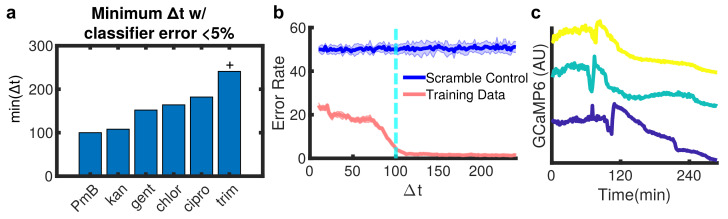
*Ca*${}^{2+}$*dynamics distinguish sensitivity and resistance to other drug classes.* (**a**) Comparing the minimum Δt to reach <5% error rates for 6 different antibiotics. Abbreviations are: PmB = Polymyxin B, kan = Kanamycin, gent = Gentamicin, chlor = Chloramphenicol, cipro = Ciprofloxacin, trim = Trimethoprim. See Figure S7, for error traces and full-time course classifier performance for all drugs. See Table S1 for concentrations tested as well as optimal hyper-parameters for each classifier. Plus-sign indicates that Trimethoprim never reaches <5% error threshold in 240 minute time-course. (**b**) Error rate as a function of Δt for Polymyxin B sensitivity classifier (red curves). The blue curve is the error rate of the same random forest architecture trained on scrambled cell labels. Error bars are the variance calculated between 10 and folds (80/20 split). Dashed, cyan line marks when the error rate is first below 5% at 100 min. (**c**) Example GCaMP6 signal in three single cells in response to 10 μg/mL Polymyxin B.

Given the accuracy of the classifiers in predicting kanamycin treatment, we were next interested in investigating if disruption of Ca${}^{2+}$ homeostasis was an early marker of response to other antibiotics. Using our imaging and machine learning framework, we measured the Ca${}^{2+}$ dynamics in *E. coli* in response to 5 other antibiotic compounds spanning several drug classes (Figure 7a). Distinct random forest classifiers were generated for each new compound. We used the same threshold (minimum time to <5% error) to compare between different drugs’ classifiers. We found a wide range of temporal dependencies for the different drugs ranging from <100 min (Polymyxin B) to never reaching the 5% threshold (Trimethoprim) (Figure 7a, Figure S7). Interestingly, the hyper-parameters on the maximum number of tree splits and minimum leaf size, both measures of classifier complexity, modestly related to the predictive time suggesting classifier complexity scaled proportionally to the information contained in the traces (Table S1).

We were particularly interested in Polymyxin B, as the temporal dependence on classifier predictivity was especially pronounced (Figure 7b). The Polymyxin B classifier was more predictive than the kanamycin classifier with an error rate of <1% for both sensitive and resistant classification (Figure S7a). This was remarkable given the extensive connection between Ca${}^{2+}$, membrane voltage, and its role in mediating aminoglycoside sensitivity [35]. Polymyxin B is a chimeric, non-ribosomal peptide originally derived from *Bacillus polymyxa* [45]. A zwitterionic compound, one end displaces Ca${}^{2+}$ and Mg${}^{2+}$ in the outer membrane matrix, binding the lipopolysaccharides (LPS) of Gram negative bacteria, while the other end acts as a detergent, permeabilizing the lipid bilayer [46]. A 1970s study found that increasing the concentrations of Ca${}^{2+}$ decreased the potency of Polymyxin B in bacterial cultures [47], and the importance of cation concentrations for reliable antimicrobial susceptibility measurements for colistin and Polymyxin B has been previously discussed [48,49]. Ca${}^{2+}$ is thought to reduce binding of Polymyxin B, which competes with Ca${}^{2+}$ to bind the LPS in Gram-negative cells. Additionally, a seemingly unrelated observation found CCCP, an ionophore, to sensitize resistant cells to Polymyxin B [50,51]. This was proposed to be mediated by a restoration of the negative charge on the LPS via disruption of the proton motor force. Therefore, circumstantial evidence ties Polymyxin B mechanism to membrane voltage.

**Figure 8:**
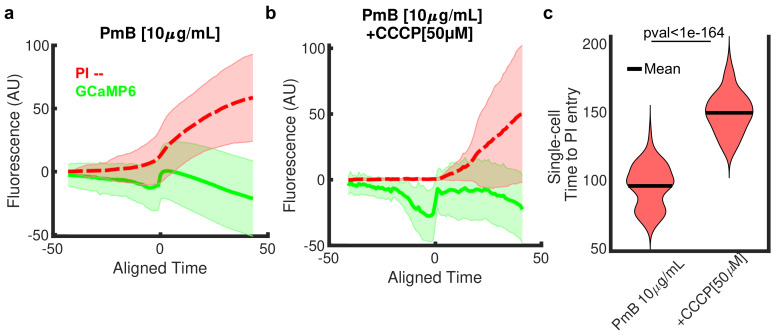
*Polymyxin B induces three-phase Ca*${}^{2+}$*dynamics.* (**a**) Time aligned GCaMP6 (green curve) and propidium iodide (PI, red dashed curve) traces in 10 μg/mL Polymyxin B (PmB). Because the Ca${}^{2+}$ blink does not happen simultaneously in all cells, we aligned the traces to the last time-point where the derivative of the GcAMP6 signal is 5 standard deviations above average after 150 min (Figure S8a). This point is Aligned Time = 0 in the plot. The GCaMP6 and PI signals from −45 to +45 min of this point are included. Fluorescence is normalized to the average intensity of the signal between frames 10 and 20. (**b**) The GCaMP6 and PI traces with the addition of 50 M CCCP in 10 μg/mL PmB treatment condition. (**c**) The distribution of time to PI entry in single cells for 10 μg/mL PmB treatment with or without CCCP. PI entry was defined as the fluorescent signal 5 standard deviations above the mean signal over the first 40 frames. The p-value was calculated using a one-tail Mann–Whitney U test (null hypothesis: Median(+CCCP)<Median(-CCCP)). The presence of CCCP increases the mean time to PI entry (black lines) from 95 to 149 min. See Video S3.

When we examined the GCaMP6 signal in Polymyxin B (Figure 1c), we observed a pronounced Ca${}^{2+}$ blink (Figure 7c, Video S3), in contrast to the Ca${}^{2+}$ transients characteristic of kanamycin. Aligning the single-cell blinks (Figure S8) revealed Ca${}^{2+}$ has three phases in response to Polymyxin B (Figure 8a, green curve). Initially, free Ca${}^{2+}$ decreases in the cytosol. This phase is followed by a large and rapid spike in GCaMP6 signal, which we termed a blink. Finally, the amount of free Ca${}^{2+}$ decayed to background concentrations as the external and internal Ca${}^{2+}$ equilibrated. The initial decline in Ca${}^{2+}$ precedes the entry of propidium iodide (PI, a measure of membrane permeability) into the cell (Figure 8a, red curve, Video S3) suggesting the initial loss of Ca${}^{2+}$ in the cytosol is mediated by the change in periplasmic [Ca${}^{2+}$]. In support of this, we found the phases of Ca${}^{2+}$ response depended on the concentration of external Ca${}^{2+}$ (Figure S9a,b). Additionally, the onset time of PI entry was dose-dependent (Figure S10a), but the time between the Ca${}^{2+}$-blink and PI entry was not (Figure S10b).

The phases of Ca${}^{2+}$ response also depended on membrane voltage (Figure 8b, Figure S9c,d). Dissipating the membrane voltage using CCCP magnified the initial decline in Ca${}^{2+}$ as well as the subsequent blink (Figure 8b). Interestingly, despite CCCP sensitizing cells [50,51], the presence of CCCP delayed the time to PI entry in the cells by nearly 66% (Figure 8c, Video S3). Delayed PI uptake in the absence of membrane voltage is not a result of changes in electrostatic-amplified diffusion of PI as PI has a neutral formal charge. This suggests membrane voltage modifies the rate of membrane permeabilization with Polymyxin B. Finally, we found external Ca${}^{2+}$ modified both the absolute time to PI entry and the time between the GCaMP6 blink and the entry of PI (Figure S10c–e) suggesting that Ca${}^{2+}$ flux plays a previously unappreciated role in the membrane break down.

## 4. Discussion

All cells live on a razor edge with respect to Ca${}^{2+}$. On one side, they must maintain a 4-order of magnitude gradient between the extracellular environment and the cytosol to prevent cytotoxicity. On the other side, precise Ca${}^{2+}$ release in space and time is required control cell signaling. This dichotomy has been well studied in eukaryotes; however, limited tools exist for modifying prokaryotic Ca${}^{2+}$. In this work, we combined fluorescent Ca${}^{2+}$ imaging with machine learning algorithms to uncover new facets of Ca${}^{2+}$ signaling in *E. coli* and *B. subtilis*. Our approach provided a unique view of single-cell pharmacokinetics and pharmacodynamics (Figure 6) revealing the dose-dependent contributions of heterogeneous drug response and time to onset on the bactericidal activity of kanamycin. While the dynamic range of the machine learning framework is significantly lower than the CFU assay (~3/100 versus $1/10^{9}$, respectively) the strong dependence of drug action on dose and time is apparent even at the single-cell level (Figure 6). However, the relationship between the time point at which a cell is correctly labeled as sensitive to the actual time-point that cell is no longer viable is unknown. The use of pulse-chase experiments will be helpful to determine this relationship. Additionally, our approach does not rule out Ca${}^{2+}$ dynamics as a signal of antibiotic activity for drugs which took longer for the random forest to classify (Figure 7a), merely that time to onset of such disruption is delayed compared to Polymyxin B and kanamycin. Finally, our model does not currently distinguish between tolerant, persistent, and resistant cell types, which are important distinctions clinically [52,53]. We envision with specific cell markers and enough training data, these sub-states could also be distinguished by machine learning algorithms at single-cell resolution.

As prefaced above, one of the foremost challenges in the study of Ca${}^{2+}$ in prokaryotic organisms is the dearth of tools to directly manipulate free Ca${}^{2+}$ due to the large stores in the periplasm [5,54]. Depleting or overloading Ca${}^{2+}$ in the extracellular environment is readily compensated by the cell [54]. The paucity of approaches to controlling intracellular Ca${}^{2+}$ has precluded identifying the function of the many predicted Ca${}^{2+}$-binding proteins [20]. The discovery of Ca${}^{2+}$-mediated signaling processes in prokaryotes, akin to proteases and phospholipases in eukaryotes, depends on the ability to directly modulate free Ca${}^{2+}$ inside the cell. Here, we have investigated how Ca${}^{2+}$ dynamics differentiate sensitive and resistant cells in the context of antibiotic treatment and found that Ca${}^{2+}$ dynamics contain rich information regarding cellular stresses. While the induced changes in Ca${}^{2+}$ are not the classic dose–response relationship, titrating intracellular Ca${}^{2+}$ proportional to the input, each perturbation represents a reproducible change in Ca${}^{2+}$ homeostasis. Leveraging an expanding arsenal of reproducible perturbations could be combined to enable a systems control framework for manipulating Ca${}^{2+}$ inside the cells. As we observed, the dose and timing are critical for the onset of distinguishing Ca${}^{2+}$ dynamics in the aminoglycosides and Polymyxin B, suggesting that timing sample collection will be critical for identifying Ca${}^{2+}$-dependent processes.

Our studies suggest a novel, voltage enhanced breakdown of membrane integrity during poration by Polymyxin B. Further studies are required to disentangle the role of Ca${}^{2+}$ in modifying Polymyxin B binding from voltage-mediated membrane disruption; however, our studies suggest that Ca${}^{2+}$ modifies the rate at which membrane breakdown occurs. The prototypical example of a Ca${}^{2+}$-dependent antibiotic is daptomycin [40,55], a broad-spectrum antibiotic against Gram-negative pathogens, originally derived from *Streptomyces roseosporus* [56,57]. Like daptomycin, Polymyxin B belongs to the same family of N-acylated cyclic peptides, but evolved in a different bacterial phylum. The growing body of Ca${}^{2+}$-dependent antibiotics, including the recent discovery of malacidins [40], suggests a broader role of disruption of Ca${}^{2+}$ homeostasis in mediating cell death. Given the paradox Ca${}^{2+}$ presents to cells, being both necessary and cytotoxic, it is reasonable to suspect that bacterial assailants have evolved many mechanisms to manipulate their neighbor’s Ca${}^{2+}$. In eukaryotes, Ca${}^{2+}$ connects membrane voltage to cellular signaling; therefore, it further seems plausible that the existence of such mechanisms in prokaryotes would provide ample opportunity for the development of many Ca${}^{2+}$-dependent natural products in both commensal and amensal relationships.

One immediate application of this work would be to improve the rapid detection of antibiotic sensitivity in clinical samples. Machine learning approaches have been developed to accelerate classic antibiotic sensitivity tests (AST) by increasing the detectable changes in growth in the presence of antibiotic [58,59,60,61]. Two notable clinical products of these efforts are the Accelerate Pheno and the Vivek 2 [62,63], clinically approved instruments which automate AST. However, the time to diagnosis remains approximately 8 h after an overnight culture, while antibiotics are commonly prescribed immediately. The insight that Ca${}^{2+}$ is a reliable and early indicator of antibiotic sensitivity could be leveraged to further increase the speed of these algorithms instead of waiting for gross phenotypic changes such as cell growth. That this could be done at single-cell resolution opens the possibility of examining mixed populations to estimate the prevalence of resistant cells. While we demonstrated our approach was applicable to multiple species (*E. coli* and *B. subtilis*), and multiple antibiotic classes (aminoglycosides, quinolones, and colistins), our approach also necessitates building specific classifiers for each pair. By building a library of these classifiers, specific to pairs of species and antibiotics, rapid detection of antibiotic resistance in bacterial consortia could be achieved. An additional limitation of our approach is the requirement of stable expression of genetically-encoded, fluorescent sensors. This limitation could be overcome by leveraging Nernstian dyes or other live-cell, staining-based measurements of cellular physiology which changes in response to antibiotic stress. For example, stains which measure changes in DNA accessibility in response to beta-lactamases [64] could be explored. Integrating multiple signals of dynamic physiology, akin to a dynamic version of cell painting [65] developed for eukaryotic cells, will further enrich our understanding of drug mechanisms of action while simultaneously increasing the speed at which computers can identify resistant pathogens in the clinic.

## Data Availability

All code and processed data required to create and train the models are available from github at the following address https://meyerct1@bitbucket.org/meyerct1/gcamp_paper.git accessible as of 1 May 2021. Raw images and image processing code are available upon request to lead author.

## Supplementary Materials

The following are available online at https://www.mdpi.com/article/10.3390/microorganisms9051000/s1.
