# Supplementary material for: Machine Learning Establishes Single-Cell Calcium Dynamics as an Early Indicator of Antibiotic Response
Source: Microorganisms. 2021 May 5;9(5):1000. doi: 10.3390/microorganisms9051000 (PMC8148219; doi:10.3390/microorganisms9051000)
Supplement: Supplementary file 1 [file microorganisms-09-01000-s001.zip › microorganisms-1170637-supplementary.pdf]

Machine Learning Establishes Single-Cell Calcium Dynamics as an Early Indicator of Antibiotic Response

Christian T. Meyer[1], Megan P. Jewel[1], Eugene J. Miller[1], and Joel M. Kralj[1 *]

[1]BioFrontiers and MCDB Department, University of Colorado Boulder, Boulder, CO 80303, USA;
[*]Correspondence should be directed to J.M.K. (joel.kralj@colorado.edu).

Supplemental Materials

Supplemental Figures

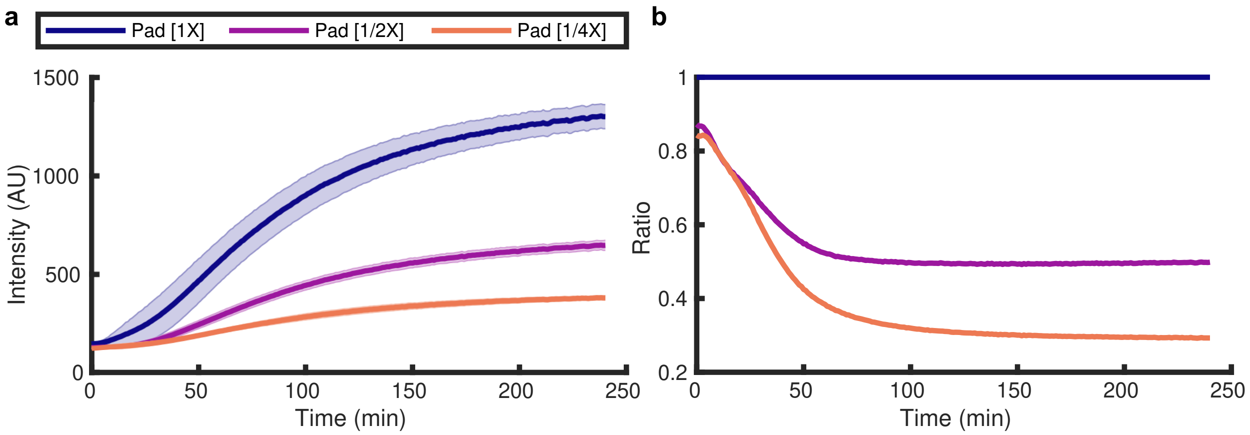

Figure S1: *Pharmacokinetics of drug delivery in vitro.* **(a)** Measurement of fluorescein dye diffusion through agar pad over 4 hour time-course. 5μL of 40X concentration of dye was added on top of 200μL pad immediately prior to commencing imaging. 1:2 dilution series is shown. Minimal variation between wells (3 wells/condition) and between location in the well (3 locations/well) is observed (shaded error bars). **(b)** The dilution series converge to 1:2 (purple) and 1:4 (orange) of maximum concentration within the first 100 minutes.

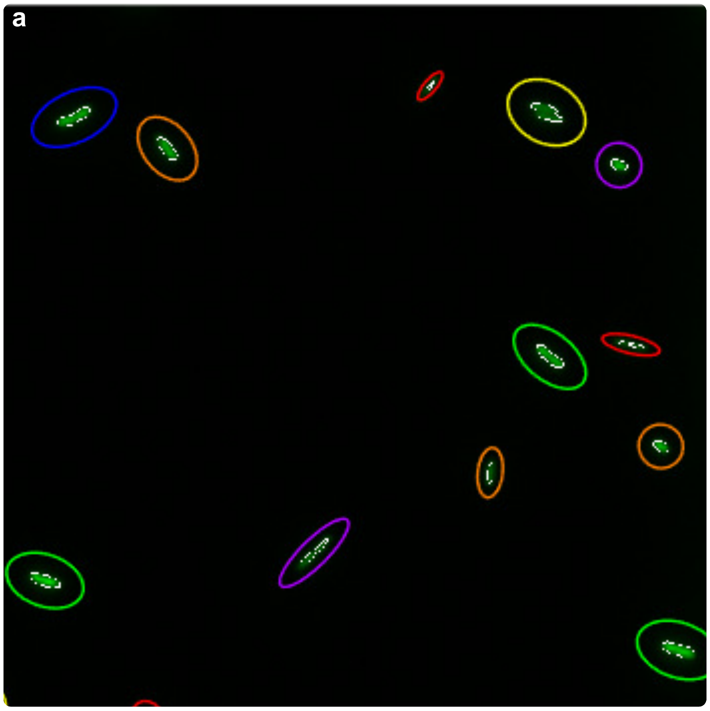

Figure S2: *Elliptic segmentation.* **(a)** After cells were segmented using a Hessian-based algorithm (white outline, see Methods), an ellipse with major and minor axes of 1X and 1.5X segmented object length and width was used to define the region of Ca^{2+} traces are extracted from. Only pixels above background within each ellipse contribute to the summary statistics and cell growth calculations. This allows for small amounts of drift and cell growth over the course of the 4 hours (See Video S1).

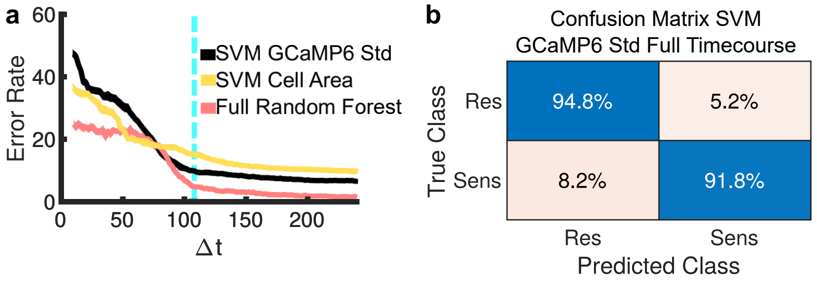

Figure S3: *Including additional features in Ca^{2+} dynamics beyond Ca^{2+} transients (GCaMP6 std) improves the classification of sensitive and resistant cells necessitating a random forest classifier.* **(a)** Comparing the error rate as a function of Δt between a simple statistical classifier (SVM) based only on Ca^{2+} transients (*i.e.* GCaMP6 std, black line) to the full random forest classifier (pink line, matches trace in Figure 5a). Also plotted is the classifier performance of the SVM trained on cell area (yellow, matches trace in Figure 4a). **(b)** The confusion matrix of the SVM based on the GCaMP std. While the SVM classifier trained on GCaMP6 std outperforms the SVM based on cell area, including all the calculated features in an albeit more complex random forest classifier substantially improves the overall predictivity and the speed at which this predictivity is achieved.

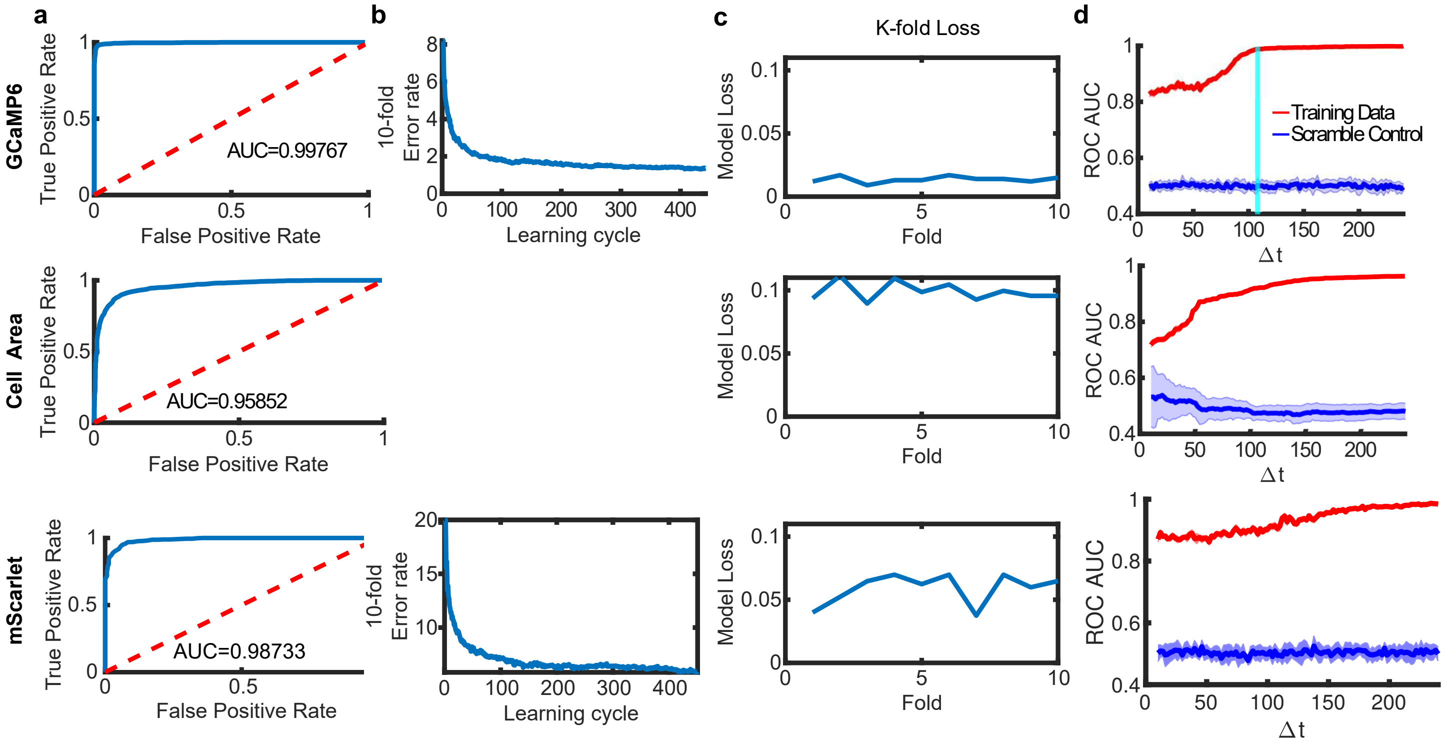

Figure S4: *Random forest classifier performance for GCaMP6, cell area, and mScarlet signal in kanamycin treated samples.* **(a)** Receiver Operating Curve (ROC) for the full-time course classifiers (Δt=241min, blue line). As the threshold for false-positive rate increases, the true positive rate should approach 1. Poor classifiers fall along the diagonal (red dotted line) corresponding to a linear increase in the number of false positives as the true positive rate is increased. Optimal classifiers approach a square meaning 100% true positive rate at a low false-positive rate. For such classifiers, the area under the ROC curve (AUC) approaches 1. **(b)** The 10-fold error rate as a function of the number of learning cycles. Number cycles based on hyperparameter optimization determined > 400 cycles optimal for both GCaMP6 and mScarlet signals. This can be observed in the plateau in the error rate as the learning cycles exceeds 400. The SVM used for classifying based on cell area does not include learning cycles. **(c)** After training, the magnitude of and variance between the loss for 10-fold cross-validation is smaller for GCaMP6 signal than cell area and mScarlet. The small variance between folds indicates the classifier is not overfitting the data. **(d)** As Δt increases, the classifiers' ROC AUC increases. Cyan line marks when the classifiers reach $< 5\%$ error rate.

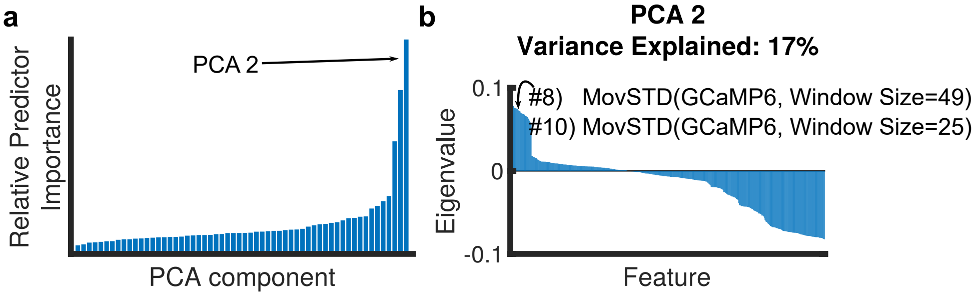

Figure S5: *Highly predictive features in the Ca^{2+}-based random forest for kanamycin treatment includes Ca^{2+} transients.* **(a)** The relative predictor importance for the 58 PCA components used to train the full time-course kanamycin classifier in *E. coli*. Feature importance calculated using Matlab's *predictorImportance* function. PCA2 was the most important component in discriminating between sensitive and resistant cells. **(b)** Waterfall chart for PCA2 eigenvalues. The most important features in PCA2, which described 17% percent of the total variance in the dataset, include moving window standard deviation of the mean GCaMP6 signal. This was the original definition of Ca^{2+} transients (see Figures 2c, 3, 4) used to distinguish sensitive and resistant cells.

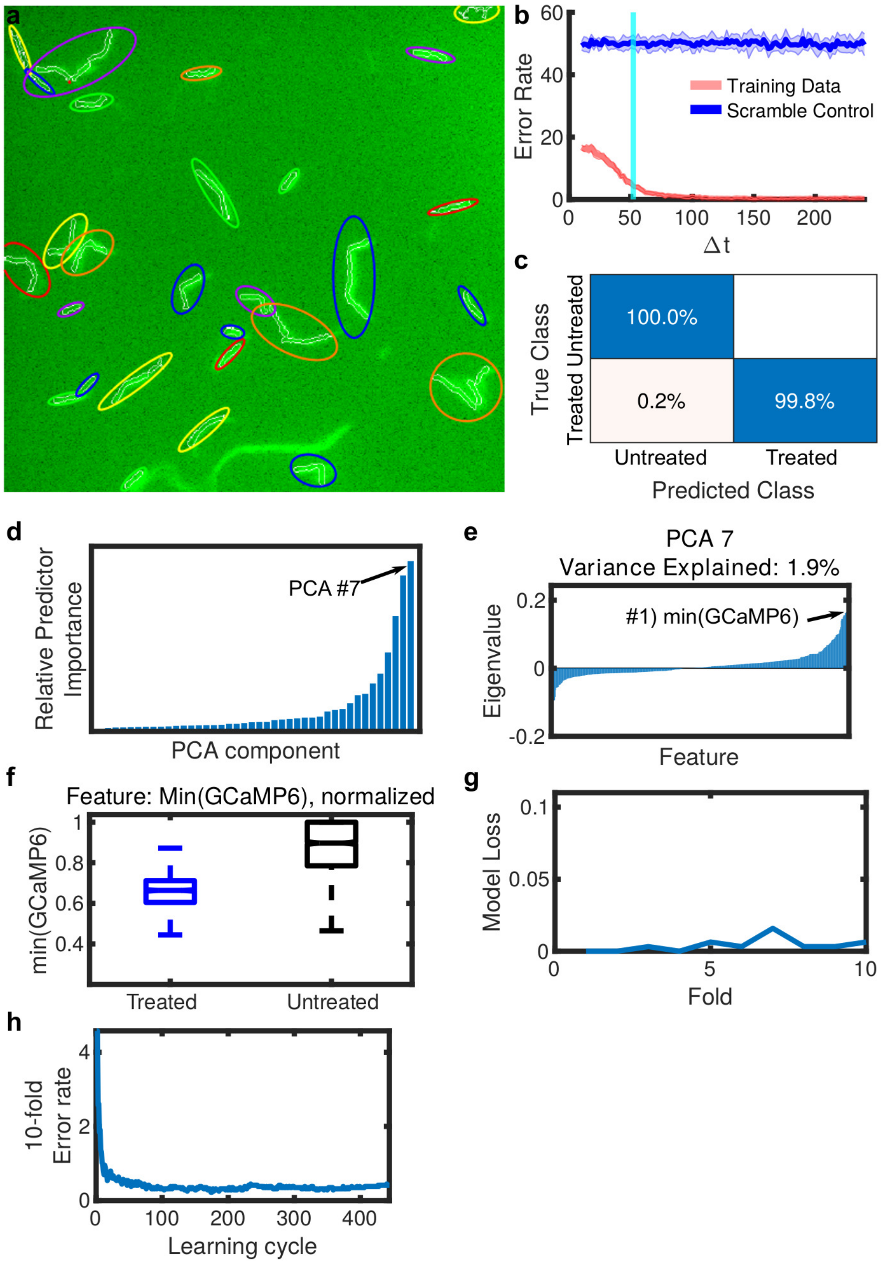

Figure S6: *Distinguishing kanamycin treated B. subtilis using Ca^{2+} dynamics.* **(a)** Image of *B. subtilis* with genomically-incorporated GCaMP6 (IPTG-inducible hyperspank promoter). Elliptic segmentation overlaid. Due to the formation of chains and the lower GCaMP6 expression, the segmentation does not separate single cells as well as for *E. coli* (Figure S2). **(b)** Error rate of the random forest classifiers as a function of Δt. Cyan line marks where the classifiers first reach $< 5\%$ error rate (52 minutes). Shaded error bars are the standard deviation in the error rate between 10-folds (80/20 split). **(c)** Confusion matrix for the full time course classifier distinguishing treated and untreated *B. subtilis* cells based on GCaMP6 signal. 100% of the untreated cells are correctly classified after 4 hours. **(d),(e)** The most important predictor used by the full time course classifier was PCA7 despite containing only 1.9% of the total variance in the dataset. The feature with the largest eigenvalue in PCA7 is the minimum of the cells' GCaMP6 signal over time. **(f)** Examining the distribution of minimum GCaMP6 signal for treated (blue boxplot) and untreated (black boxplot) cells reveals a significant difference (p-val$< 1e-10$). **(g),(h)** The classifier loss between different folds in cross validation (h) and the error rate as a function of learning cycle (g) shows the full time course classifier is not being over trained.

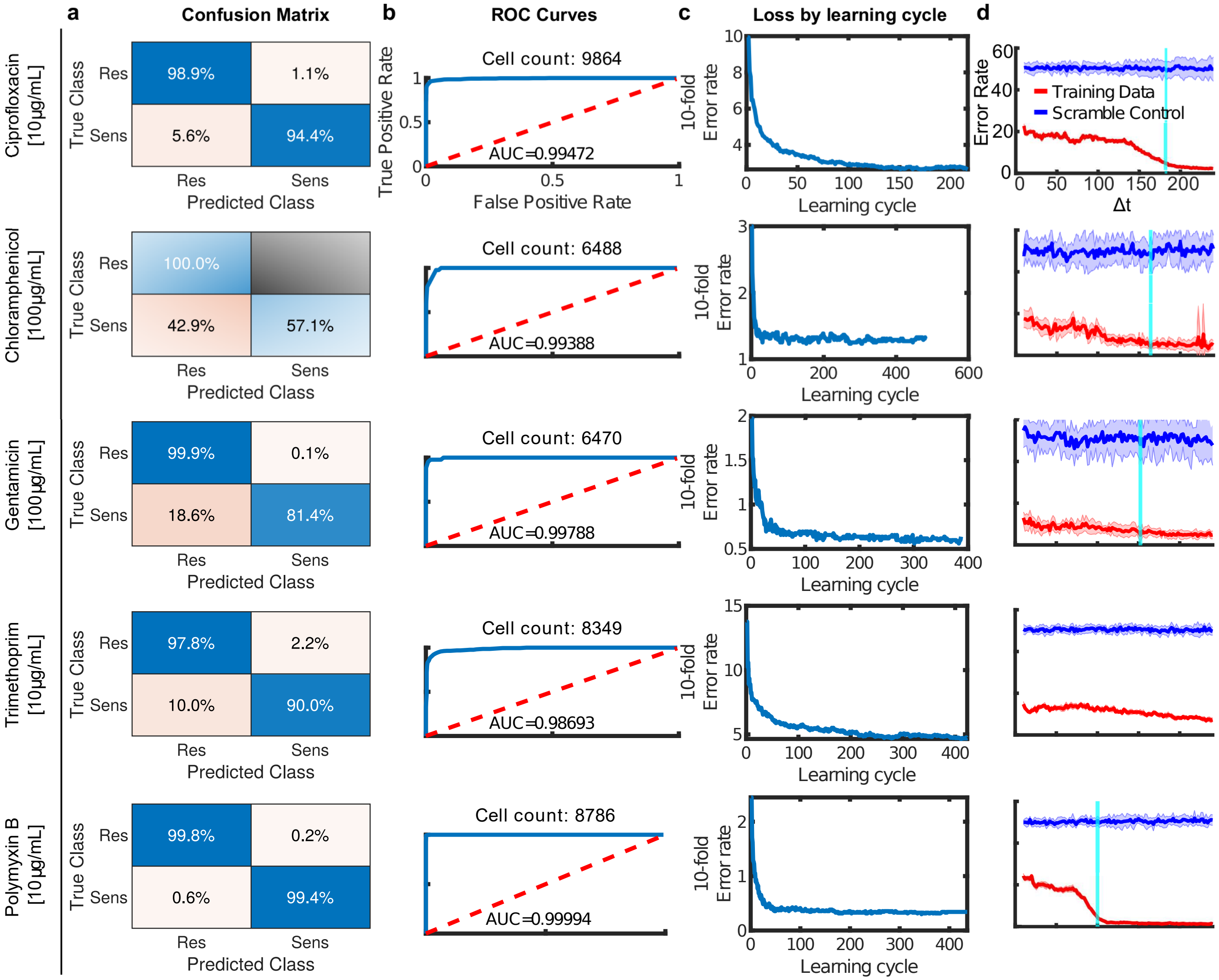

Figure S7: *Ca^{2+} dynamics in drug action for 5 different antibiotics.* **(a)** Confusion matrix of the full-time-course classifiers trained on Ca^{2+} traces for each drug. A confusion matrix was generated on 20% of the data withheld from the initial training which used 80/20, 10-fold cross-validation. **(b)** ROC curves for each drug's full-time-course classifier. **(c)** Learning cycles for each drug classifier. **(d)** Error rate as a function of Δt for each drug. All axes are the same. Cyan-line marks when classifiers reach $< 5\%$ error in single-cell classification.

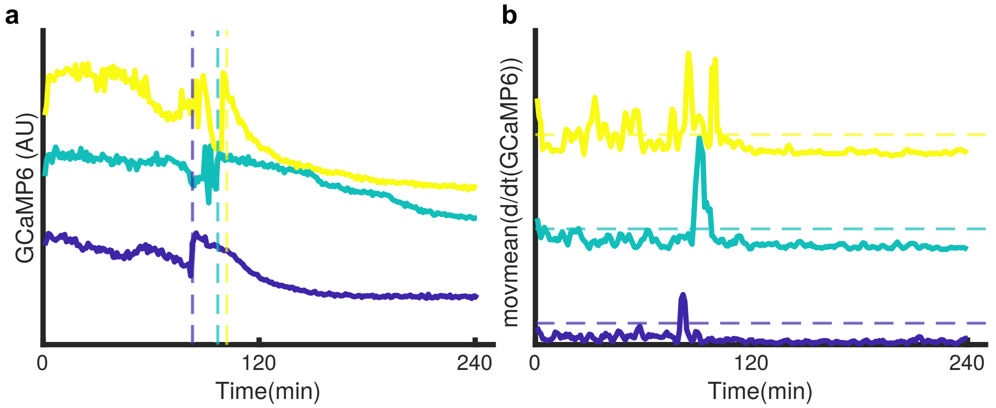

Figure S8: *Aligning GCaMP6 blinks in Polymyxin B treated cells.* **(a)** Shown are three GCaMP6 traces for single cells from the 10µg/mL Polymyxin B treatment. A blink is defined as the last point in the 3-frame, moving average of the derivative greater than 5 standard deviations above the mean derivative after 150 minutes. The vertical dashed line (color-matched) is the point the algorithm found as the last point the derivative exceeded this threshold. **(b)** The 3-frame, moving average of the GCaMP6 signal derivative. The horizontal dotted line indicates the threshold for each trace.

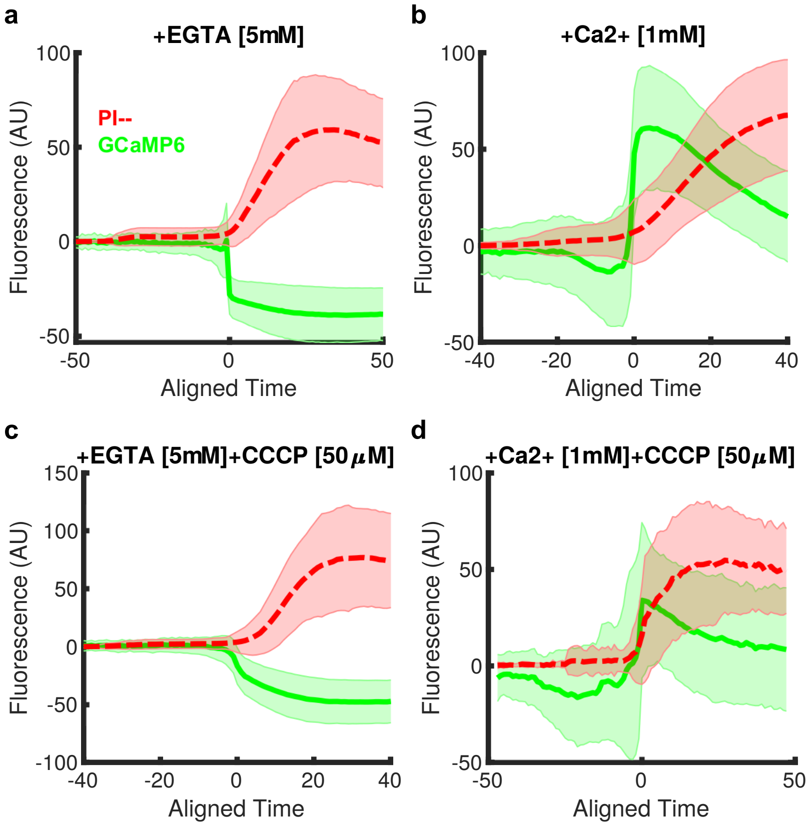

Figure S9: *External Ca^{2+} modifies the magnitude and duration of the Polymyxin B-induced blink.* **(a),(b),(c),(d)** Time aligned average GCaMP6 (green line) and PI (red dashed line) traces from single cells when treated with Polymyxin B (10µg/mL) and EGTA (a), Ca^{2+} (b), and each with CCCP (c,d). See Figure S8 for description of time alignment. The EGTA, Ca^{2+}, PI, and CCCP was added to the liquid agarose before pouring mold. This allowed the cells time to equilibrate (> 1hr) before seeing the Polymyxin B. Both the duration and magnitude of the two-phases depend on external Ca^{2+} as well as membrane potential (dissipated by CCCP). The standard PMM media has 3.6 µM free Ca^{2+}. In 5mM EGTA at pH 7.5 and 30°C, there are <1e-11 free Ca^{2+} ions per mL (See Methods for calculation).

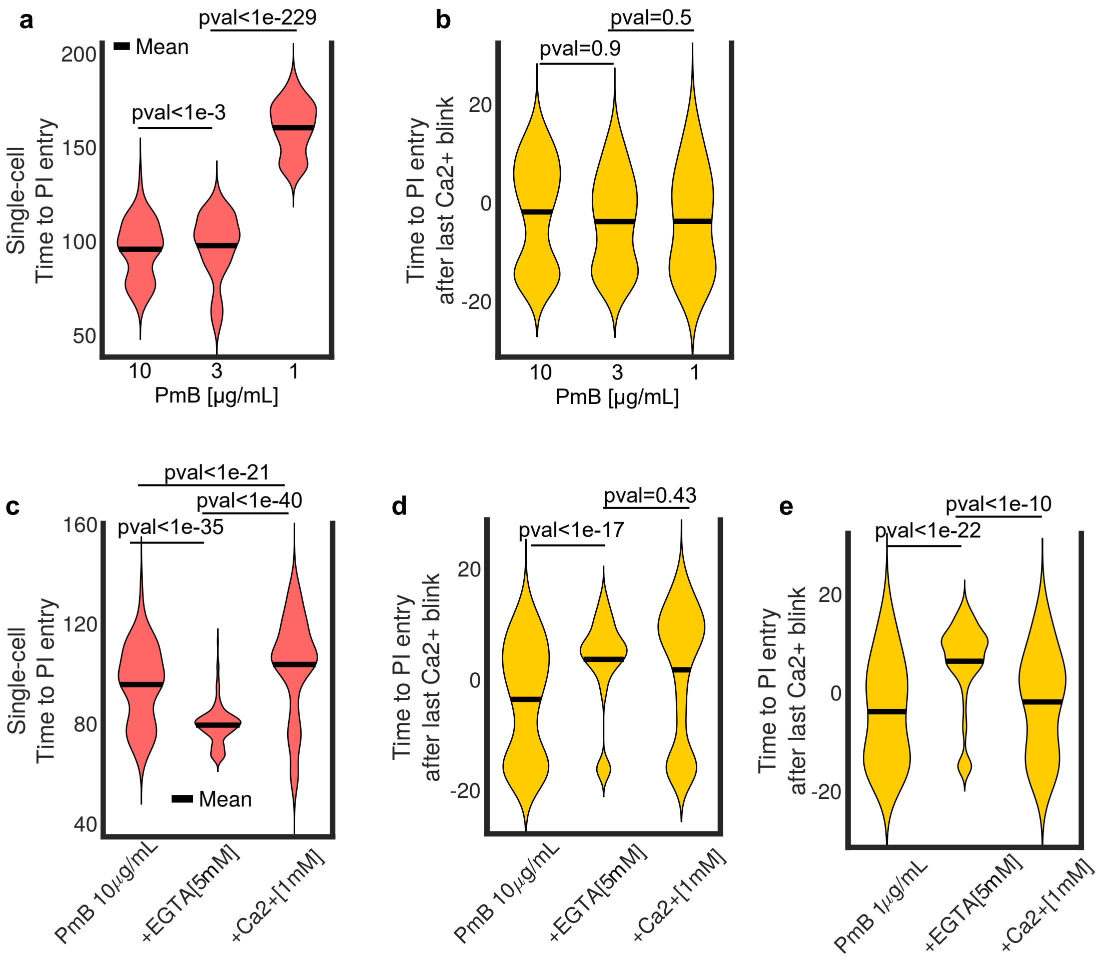

Figure S10: *External Ca^{2+} modifies the time for Polymyxin B induced membrane destabilization.* **(a)** The mean time to PI entry in the cell as a function of Polymyxin B dose. Shown are the single-cell distributions for time for PI entry. PI entry was defined as signal 5 standard deviations above the mean signal calculated over the first 40 frames. The p-values were calculated using a one-tail Mann–Whitney U test. The null hypothesis for both tests was Median(Lower PmB Concentration)>Median(Higher PmB Concentration). The black line indicates the mean. **(b)** Time to PI entry after Ca^{2+} blink. Ca^{2+} blink as defined in Figure S8. p-value calculated as in panel a. The time to PI entry after last blink does not change between different doses. **(c)** The presence of EGTA in the pad decreases the time to PI entry in the cell, consistent with the classifier of Ca^{2+} as a competitive binder with Polymyxin B in the LPS. Correspondingly, increasing Ca^{2+} in the pad increases the time to PI entry. p-value calculated using one-tail Mann-Whitney U test. The null hypotheses for these calculations in decending order: Median(PmB)>Median(+Ca2); Median(+EGTA)>Median(+Ca); and Median(+EGTA)>Median(PmB). This change mimics the effective dose trend in panel a. **(d),(e)** However, the presence of EGTA lengthens the time from the blink to the entry of PI in both 10µg/mL (d) and 1µg/mL (e) conditions. p-value calculated using one-tail Mann-Whitney U test. The null hypotheses for these calculations in decending order: Median(+EGTA)>Median(+Ca) and Median(PmB)>Median(+EGTA). This suggests the lack of a prolonged secondary spike in Ca^{2+} alters the rate of membrane poration through an unknown mechanism.

Video S1. Ellipse segmentation for simultaneously extracting GCaMP6 signal and cell growth in *E. coli*. Both fields of view are treated with 100µg/mL kanamycin. The left panel is mntH KanR cells. The right panel is sensitive BW25113. The GcAMP6 channel is shown for both panels. Images at 1 frame/minute for 240 minutes.

Video S2. Ellipse segmentation for simultaneously extracting GCaMP6 signal and cell growth in *B. subtilis*. The left panel is untreated cells. The right panel is treated 100µg/mL. The GCaMP6 channel is shown for both panels. Images at 1 frame/minute for 240 minutes.

Video S3. GCaMP6 and PI signal in Polymyxin B [10µg/mL] treated cells with and without CCCP, left and right panel, respectively. PI staining in red. GCaMP6 signal in green. Time frames match between two panels.

Table S1: Hyperparameters for the different drugs' random forest classifier. Hyperparameters optimized using the full-time-course data and Bayesian optimization using the *fitcensemble* method in Matlab. First boosting algorithm was optimized followed by hyper-parameter selection. For all classifiers, we found AdaBoostM1 boosting was preferred. 200 objective evaluations were performed during hyper-parameter selection.

Drug	Max Conc. (µg/mL)	# Learning Cycles	Learning Rate	Min Leaf Size	Max # Splits
Kan	100	442	0.94414	7	34
Cipro	10	217	0.40054	177	5519
Chlor	100	482	0.69145	23	15
Trim	10	423	0.90351	117	64
PmB	10	436	0.59427	10	58
Gent	100	387	0.82293	55	6
Kan (*B. subtilis*)	100	451	0.98779	59	21

Feature calculation from fluorescent traces

To calculate features from the Ca^{2+} traces, a mixture of convolution functions and normalization protocols are applied to different moments of the GCaMP6 fluorescence measured for each cell. Specifically, for the min, max, mean, and standard deviation of the pixels' fluorescent intensity inside the segmentation ellipse over time, the following moments are computed for a particular Δt: mean, median, std, max, and min of the zero, first and second-order derivatives. The moving mean and moving standard deviation are used as convolution filters with window sizes equal to 20% and 10% of Δt. All features are calculated again after normalizing the signal by the first time point. What follows is the Matlab-code for recreating these features.

```
%Function for calculating feature matrix of traces
function [master_data,mastLab] = calcFeatureMatrix(S,deltaT)
    %For each of the field names in the structure S (Mean/Min/Max/Std of GCaMP6 over time)
    nms = fieldnames(S);
    master_data  = []; mastLab=[]; %Feature matrix and feature labels list
    for j = 1:length(nms) %For each of the mean,min,max, and std calculate the following
        %Calculate moments using calcTrajFeatures (see below)
        [mat1,labs] = calcTrajFeatures(S.(nms{j}));
        for l=1:length(labs)                        %Keep track of labels for each feature
            labs{l} = [labs{l} '_' nms{j}];
        end
        master_data = [master_data,mat1];                %append the data
        mastLab = [mastLab,labs];
        %Calculate the features convolving by taking the mean
        %for 20% and 10% trajectory length
        for i=[5,10]
            [mat1,labs] = calcTrajFeatures(movmean(S.(nms{j}),ceil(deltaT/i),2));
            for l=1:length(labs)
                labs{l} = [labs{l} '_' nms{j} '_movmean_' num2str(ceil(deltaT/i))];
            end
            master_data = [master_data,mat1];
            mastLab = [mastLab,labs];
        end
        %Calculate the features convolving by taking std for 20% and 10% trajectory length
        for i=[5,10]
            [mat1,labs] = calcTrajFeatures(movstd(S.(nms{j}),ceil(deltaT/i),[],2));
            for l=1:length(labs)
                labs{l} = [labs{l} '_' nms{j} '_movstd__' num2str(ceil(deltaT/i))];
            end
            master_data = [master_data,mat1];
            mastLab = [mastLab,labs];
        end

        %Now normalize matrix to T==0
        %And compute same features
        tmp = S.(nms{j});tmp = tmp./tmp(:,1);
```

```
        [mat1,labs] = calcTrajFeatures(tmp);
        for l=1:length(labs)
            labs{l} = [labs{l} '_' nms{j} '_norm'];
        end
        master_data = [master_data,mat1];
        mastLab = [mastLab,labs];
        for i=[5,10]
            [mat1,labs] = calcTrajFeatures(movmean(tmp,ceil(deltaT/i),2));
            for l=1:length(labs)
                labs{l} = [labs{l} '_' nms{j} '_norm_movmean_' num2str(ceil(deltaT/i))];
            end
            master_data = [master_data,mat1];
            mastLab = [mastLab,labs];
        end
        for i=[5,10]
            [mat1,labs] = calcTrajFeatures(movstd(tmp,ceil(deltaT/i),[],2));
            for l=1:length(labs)
                labs{l} = [labs{l} '_' nms{j} '_norm_movstd_' num2str(ceil(deltaT/i))];
            end
            master_data = [master_data,mat1];
            mastLab = [mastLab,labs];
        end
    end

end

function [mat,labs] = calcTrajFeatures(A)
    men                 = mean(A,2);                    %Mean of trace
    md                  = median(A,2);                  %Median of trace
    sd                  = std(A,[],2);                  %Std of trace
    mx                  = max(A,[],2);                  %Max of trace
    mn                  = min(A,[],2);                  %Min of trace
    men_dt              = mean(diff(A,1,2),2);          %Mean of 1st derivative of trace
    md_dt               = median(diff(A,1,2),2);        %Median of 1st derivative of trace
    sd_dt               = std(diff(A,1,2),[],2);        %Std of 1st derivative of trace
    mx_dt               = max(diff(A,1,2),[],2);        %Max of 1st derivative of trace
    mn_dt               = min(diff(A,1,2),[],2);        %Min of 1st derivative of trace
    men_dtdt            = mean(diff(A,2,2),2);          %Mean of 2nd derivative of trace
    md_dtdt             = median(diff(A,2,2),2);        %Median of 2nd derivative of trace
    sd_dtdt             = std(diff(A,2,2),[],2);        %Std of 2nd derivative of trace
    mx_dtdt             = max(diff(A,2,2),[],2);        %Max of 2nd derivative of trace
    mn_dtdt             = min(diff(A,2,2),[],2);        %Min of 2nd derivative of trace
    %Append labels
    labs                = {'Mean','Median','Std','Max','Min',...
                           'dtMean','dtMedian','dtStd','dtMax','dtMin',...
                           'dtdtMean','dtdtMedian','dtdtStd','dtdtMax','dtdtMin'};
    mat                 = [men,md,sd,mx,mn,men_dt,md_dt,sd_dt,mx_dt,mn_dt,...
                          men_dtdt,md_dtdt,sd_dtdt,mx_dtdt,mn_dtdt];
end
```
